# Characteristics of tumor thrombosis in the inferior mesenteric vein from colorectal cancer: A report of three cases and review of the literature

**DOI:** 10.1016/j.ijscr.2022.107840

**Published:** 2022-12-17

**Authors:** Shota Izukawa, Hiroyuki Mushiake, Takuo Watanabe, Tetsushi Ishiguro, Kenki Segami, Tadao Fukushima

**Affiliations:** Department of Surgery, Saiseikai Yokohamashi Nanbu Hospital, Yokohama, Japan

**Keywords:** Colorectal cancer, Tumor thrombosis, Inferior mesenteric vein, Case report

## Abstract

**Introduction:**

Intravenous tumor thrombosis is a rare condition in colorectal cancer and shows a locally aggressive biological behavior. We herein report three cases of colorectal cancer with tumor thrombosis in the inferior mesenteric vein (IMV) treated by colorectal resection combined with resection of the IMV under laparoscopic surgery.

**Case presentation:**

In these three colorectal cancer patients with IMV tumor thrombus, IMV tumor thrombus was detected in all instances on preoperative computed tomography. Preoperative chemotherapy was also performed in one patient with concurrent liver metastasis. All patients underwent laparoscopic locally R0 resection; however, the pathological findings showed a positive margin for IMV resection in all patients.

**Clinical discussion:**

We reviewed 19 previously reported cases along with our 3 present cases and clarified the characteristics of colorectal cancer accompanied by IMV tumor thrombosis. IMV tumor thrombosis may be a risk factor for liver metastasis and R1 resection, and systemic treatment, including neoadjuvant chemotherapy (NAC), may be quite important.

**Conclusion:**

IMV tumor thrombosis may have a tendency to cause hematogenous metastasis. Systemic therapy, including NAC, may be useful, but since this is a rare condition, the accumulation of further cases is needed.

## Highlights

• IMV tumor thrombosis is a rare condition in CRC.
• CRC with IMV tumor thrombosis is prone to hematogenous metastasis.
• CRC with IMV tumor thrombosis may be a risk factor for R1 resection.
• Systemic chemotherapy should be considered for CRC with IMV tumor thrombosis.

## 1 Introduction

Intravenous tumor thrombosis is a relatively common pathology in some cancers, such as portal vein thrombosis from hepatocellular carcinoma and vena cava tumor thrombosis from renal cell carcinoma. However, it is rare in colorectal cancer [1].

We herein report three cases of colorectal cancer with tumor thrombosis in the IMV treated with laparoscopic surgery and combined resection of the IMV. In addition, to consider the treatment strategies, we reviewed 19 previously reported cases along with our 3 present cases and investigated the characteristics of colorectal cancer accompanied by IMV tumor thrombosis. This research work is reported in line with the SCARE 2020 criteria [2].

## 2 Case presentation

Three female patients (Patients 1, 2 and 3: 66, 67 and 77 years old) had been diagnosed with colorectal cancer with tumor thrombosis in the IMV. No patients had any comorbidity or remarkable medical history, drug history, family history or psychosocial history. Their symptoms were shortness of breath in Patient 1, lower abdominal pain and hematochezia that had persisted for six months in Patient 2 and abdominal pain and difficulty in defecation in Patient 3. Laboratory data showed an increased level of carcinoembryonic antigen (CEA) at 59.9 ng/ml in Patient 1, an increased level of carbohydrate antigen 19-9 (CA19-9) at 194.7 U/ml in Patient 2 and an increased level of CEA at 33.7 ng/ml in Patient 3.

**Fig. 1:**
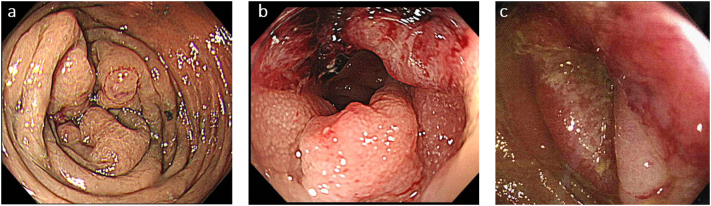
Colonoscopic findings. a) Circumferential type 3 tumor was found in the left transverse colon in Patient 1. b) Circumferential type 2 tumor was found in the rectosigmoid colon in Patient 2. c) Circumferential type 2 tumor was found in the sigmoid colon in Patient 3.

**Fig. 2:**
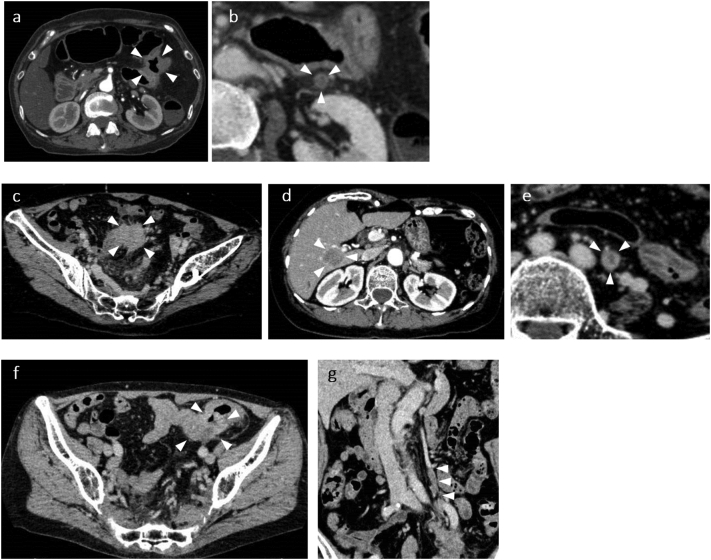
CT scan findings. a) Circumferential wall thickening with contrast enhancement was observed in the left transverse colon in Patient 1. b) IMV tumor thrombosis was suspected due to poor contrast and dilation of the IMV in Patient 1. c) Circumferential wall thickening with contrast enhancement was observed in the rectosigmoid colon in Patient 2. d) A mass lesion with irregular ring enhancement was found in liver (S6) in Patient 2. e) IMV tumor thrombosis was suspected due to poor contrast and dilation of the IMV in Patient 2. f) Circumferential wall thickening with contrast enhancement was observed in the sigmoid colon in Patient 3. g) IMV tumor thrombosis was suspected due to poor contrast and dilation of the IMV in Patient 3.

Colonoscopy showed a circumferential type 3 tumor in the left side of the transverse colon in Patient 1, a circumferential type 2 tumor in the rectosigmoid colon in Patient 2 and a circumferential type 2 tumor in the sigmoid colon in Patient 3, and the scope could not pass through due to strong stenosis in Patients 1 and 3 (Fig. 1). Abdominal contrast-enhanced computed tomography revealed an enhanced mass in the endoscopically observed lesions, and IMV tumor thrombosis was suspected due to an intraluminal filling defect in the dilated IMV in all cases. An irregular ring-enhancing lesion in segment (S) 6 of the liver was found in Patient 2 (Fig. 2).

**Fig. 3:**
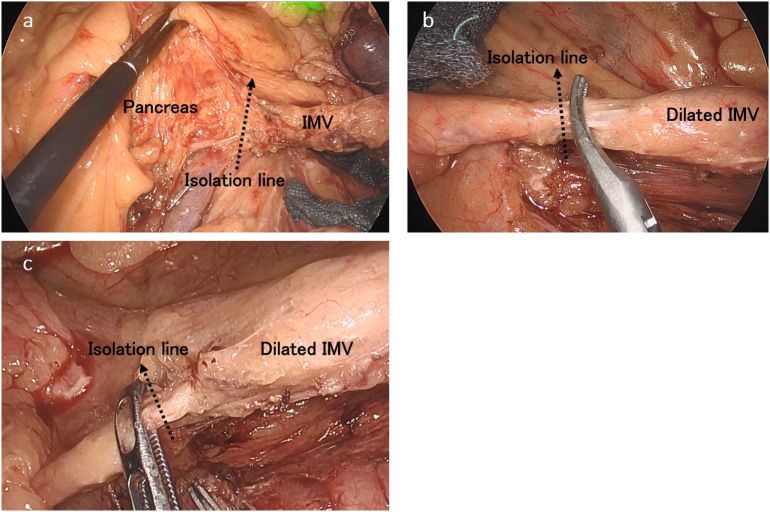
Intraoperative findings. a) We isolated the IMV at the level of the inferior margin of pancreas where the vein wall was soft and considered free from tumor invasion in Patient 1. b) We isolated the IMV at as central a point as possible to the extent that it is safely operable where the vein wall was soft and considered free from tumor invasion in Patient 2. c) We isolated the IMV at as central a point as possible to the extent that it was safely operable where the vein wall was soft and considered free from tumor invasion in Patient 3.

Patient 1 underwent laparoscopic partial colectomy of the transverse and descending colon with D3 lymphadenectomy and combined resection of the small intestine and the IMV. We performed touch confirmation of the IMV with laparoscopic forceps and identified a location where the vein wall was soft and considered free from tumor invasion. We then isolated the IMV at the level of the inferior margin of the pancreas (Fig. 3a).

Patient 2 underwent preoperative chemotherapy of modified infusional fluorouracil, leucovorin, and oxaliplatin (mFOLFOX6) plus panitumumab for four courses. The primary lesion and metastasis were reduced in size and showed a partial response to chemotherapy, so the patient was scheduled to undergo radical resection. The patient underwent laparoscopic low anterior resection with D3 lymphadenectomy and combined resection of the small intestine and IMV. We identified a location where the IMV could be isolated, as in Patient 1, and isolated the IMV at as central a point as possible to the extent that it is safely operable (Fig. 3b).

Patient 3 underwent laparoscopic sigmoid colectomy with D3 lymphadenectomy and combined resection of the retroperitoneum and IMV. We isolated the IMV as in Patient 2 (Fig. 3c).

All three surgeries were performed using the endoscopic surgical skill qualification system, involving a qualified surgeon assisted by a senior specialist general surgeon and a surgical resident. All patients had smooth postoperative courses and were discharged without major complications.

**Fig. 4:**
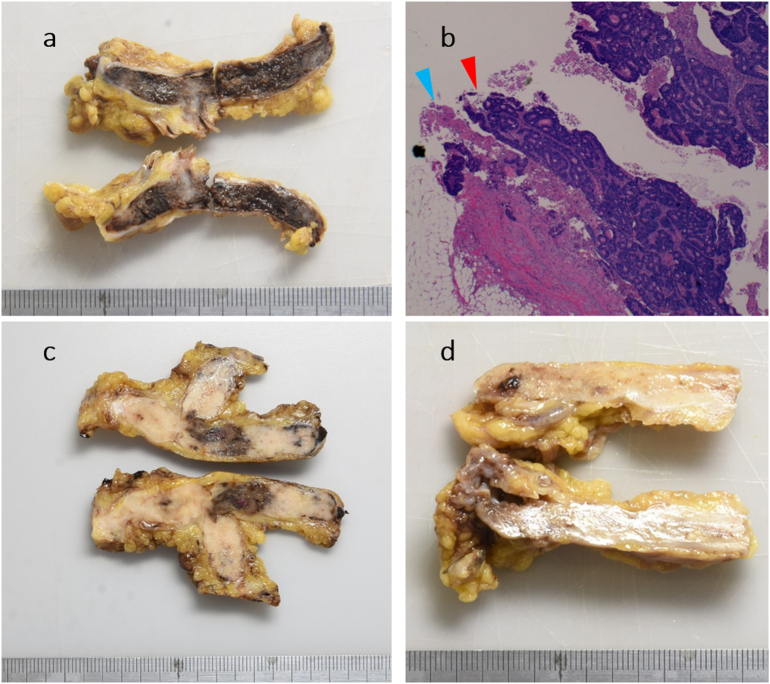
a) Macroscopic features of the resected specimen in Patient 1. Induration was palpable in the IMV, and tumor thrombosis was suspected. b) Pathological findings of the resected specimen in Patient 1. Tumor cells (red arrowhead) were found in the IMV stump (blue arrowhead), and the presence of residual cancer cells at the surgical margin was suspected. c) Macroscopic features of the resected specimen in Patient 2. d) Macroscopic features of the resected specimen in Patient 3. (For interpretation of the references to colour in this figure legend, the reader is referred to the web version of this article.)

A pathological examination showed that the tumor was a moderately differentiated adenocarcinoma in Patients 1 and 2 and a poorly differentiated adenocarcinoma in Patient 3. Lymph node metastases and lymphovascular invasion were observed in all cases. While the circumferential resection margin at the primary lesion was negative, tumor cells were found at the cut-end of the IMV, leading to the diagnosis of R1 resection in all cases (Fig. 4). The histological response to preoperative chemotherapy was Grade 1a in Patient 2. The pathological staging was T4bN1aM0, stage IIIC in Patient 1; T3N2bM1a, stage IVA (KRAS wild type, BRAF wild type) in Patient 2; and T4bN2aM0, stage IIIC in Patient 3 (8th edition of the UICC TNM classification).

Patients 1 and 3 received eight courses of capecitabine plus oxaliplatin (CAPOX) as adjuvant chemotherapy. No recurrence has been noted in the 19 months in Patient 1 and 11 months in Patient 3 since surgical resection. Patient 2 underwent right hepatic lobectomy three months after the first surgery. However, one month after this second surgery, liver metastases and multiple lung metastases were detected. mFOLFOX6 plus panitumumab was subsequently resumed, and she is currently alive 17 months after the first surgery. Adherence and tolerability to chemotherapy were found to be acceptable in all patients.

## 3 Clinical discussion

Intravenous tumor thrombosis is occasionally associated with hepatocellular carcinoma and renal cell carcinoma but is rare in colorectal cancer. Sato et al. reported that venous tumor thrombosis was detected in 3 (1.7 %) of 176 patients with advanced colorectal cancer on multidetector-row computed tomography (MDCT) [1]. Only 19 cases of IMV tumor thrombosis among colorectal cancer patients were found in a search of the pertinent literature [2], [3], [4], [5], [6], [7], [8], [9], [10], [11], [12], [13], [14], [15], [16], [17], [18]. We reviewed these 19 previously reported cases along with our 3 present cases and clarified the characteristics of colorectal cancer accompanied by IMV tumor thrombosis (Table 1).

**Table 1:** Reported cases of colorectal cancer with tumor thrombosis in the inferior mesenteric vein.

Author	Age (years)/gender	Tumor location	Tumor diameter (cm)	Pathological type	Open/lap	T	N	M	Preoperative chemotherapy	Postoperative chemotherapy	Metastasis	Outcome
Fujii	68/M	S	3.5	mod	Open	3	2	0	None	None	None	26 months survival
Deguchi	78/F	RS Ra	8	wel	Open	4a	2	0	None	None	None	48 months survival
Katsumoto	63/F	S	–	mod	Open	4a	3	1b (PALN, panc)	None	UFT/LV	synchronous (PALN, Panc)	24 months survival
Matsumoto	58/M	RS	–	mod	Lap	3	0	0	None	None	metachronous (PV)	72 months survival
Inose	75/M	S	4.5	wel	Open	4a	2	1a (liver)	None	l-LV + 5-FU → FOLFOX4	synchronous (Liver)	36 months survival
Sato	84/M	S	4	mod	–	–	–	1a (liver)	None	–	synchronous (Liver)	5.5 months death
Sato	59/M	D	8	wel	–	–	–	–	None	–	None	24 months survival
Nasu	66/M	Ra	13.5	mod	Open	3	1	0	None	mFOLFOX6	None	6 months survival
Jimi	54/F	Rab	6	muc	Open	3	0	0	None	S-1	metachronous (Liver, Lung, Adrenal gland)	50 months survival
Matsuda	64/M	Ra,S	4	mod	Open	4a	2	0	None	FOLFOX4	None	40 months survival
Ohgaki	62/F	Ra	4	mod	Open	4a	2	0	None	mFOLFOX6	None	24 months survival
Matsumura	69/F	RS	5.4	wel	Open	3	1	0	None	mFOLFOX6	metachronous (Lung)	36 months survival
Matsumura	67/M	S	5.1	wel	Lap	3	1	1a (liver)	None	mFOLFOX6 + BEV	synchronous (Liver)	7 months survival
Hasimoto	71/M	D	8	mod	Open	4b	1	0	None	mFOLFOX6	metachronous (Liver)	28 months death
Nagasue	68/M	RS	1.5	mod	Open	3	2	0	None	CAPOX+BEV	None	22 months survival
Tsukahara	64/F	D	6.2	mod	Open	4a	2	1a (PALN)	None	mFOLFOX6	synchronous (PALN)	52 months survival
Nishimura	74/M	D	5.6	mod	Open	3	1	1a (liver)	mFOLFOX6 + PANI	CAPOX	synchronous (Liver)	14 months survival
Muto	90/F	S	–	mod	–	–	–	–	–	–	–	–
Toyofuku	65/M	RS	4	mod	Lap	4a	1	0	None	mFOLFOX6	None	1 month survival
Our case	66/F	T	4	mod	Lap	4b	1b	0	None	CAPOX	None	19 months survival
Our case	67/F	RS	4	mod	Lap	3	2b	1a (liver)	mFOLFOX6 + PANI	mFOLFOX6 + PANI	synchronous (Liver)	17 months survival
Our case	77/F	S	5	por	Lap	4b	2a	0	None	CAPOX	None	11 months survival

The localization of the primary tumor was the left transverse colon in one case, the descending colon in four cases, the sigmoid colon in seven cases, the rectosigmoid in six cases, and the upper rectum in four cases, all showing primary lesions in the IMV drainage area. The histological types were highly differentiated adenocarcinoma in 5 cases, moderately differentiated adenocarcinoma in 15 cases, poorly differentiated adenocarcinoma in 1 case, and mucinous carcinoma in 1 case, with most cases being highly and moderately differentiated adenocarcinomas, showing a similar tendency to usual colorectal cancer.

In the cases for which pathology results were available, the mean tumor diameter was 54.8 mm, all cases had T3 or T4 depth and positive vascular invasion, and 17 of the 19 cases had positive lymph node metastasis. The isolating location of the IMV varied, including the root of the IMV, at the level of the inferior margin of pancreas and the confluence of the portal vein and IMV, in addition to other points. In some cases, an intraoperative rapid pathological diagnosis, vasectomy for tumor removal and cleaning were performed. Laparoscopic surgery was performed in six cases, including our three cases. Although laparoscopic surgery for colorectal cancer has been widely used in recent years and become a standard procedure in more advanced cases, it may be inferior to open surgery due to the lack of tactile sensation, and there is some concern that R1 resection may be performed in cases with intravenous tumor thrombosis. Therefore, it was considered necessary to select the surgical technique carefully.

Among the 22 total cases, 11 showed synchronous or metachronous metastasis, of which liver metastasis was the most common, occurring in 7 cases. This suggests that colorectal cancer with IMV tumor thrombosis should be considered a highly advanced status and prone to vascular metastasis. Since such extensive vascular invasion is certainly suggestive of at least micro-metastatic spread, it may be worth considering prioritizing chemotherapy as a treatment option. While postoperative adjuvant chemotherapy was administered in 16 of the 19 patients, preoperative chemotherapy was administered in only 2 patients, including one of our patients. Both cases had synchronous liver metastases, and the chemotherapy was initiated as a systemic therapy for stage IV colorectal cancer, not as NAC.

NAC has an established role in many solid tumors, including rectal cancer, but its utility has not previously been formally evaluated in colon cancer. In recent years, there have been many reports on the mid- to long-term results of NAC for colon cancer [19], [20]. In the FOxTROT trial, the R0 resection rate was 95.2 % in the NAC group and 90.9 % in the surgery-precedent group (*p* = 0.01), showing a significantly better rate in the NAC group. The 22 cases we reviewed are not identical to those in the FOxTROT trial, so we cannot say anything definitive, but they all had highly locally invasive cancer, so it is quite possible that NAC may be an effective treatment option for R0 resection. Prioritizing chemotherapy may not only increase the R0 resection rate but also potentially spare the patient pointless surgery by treating micro-metastases and allowing time for the development of extensive metastatic lesions that were not clear at the time of the initial evaluation.

## 4 Conclusions

Since colorectal cancer with IMV thrombus showed aggressive behavior and a tendency to develop liver metastasis, systemic treatment can thus be considered instead of simply assuming that radical resection is possible before surgery. Colorectal cancer with IMV thrombus is a rare condition, so the accumulation of more cases in retrospective observational studies is needed, even if randomized controlled trials are difficult to perform. These findings suggest that NAC may become a useful systemic treatment option in the future.

## Provenance and peer review

Not commissioned, externally peer-reviewed.

## Consent

Written informed consents were obtained from three patients for publication of this case report and accompanying images. A copy of the written consent is available for review by the Editor-in-Chief of this journal on request.

## Funding

This research does not receive any specific grant from funding agencies in the public, commercial, or not-for-profit sectors.

## Ethical approval

This study was approved by the Institutional Review Board (reference number 2019-D34) of Saiseikai Yokohamashi Nanbu Hospital.

## Author contribution

Substantial contributions to conception and design by Shota Izukawa MD and Hiroyuki Mushiake MD, PhD.

Acquisition of data by Shota Izukawa MD.

Drafting the article by Shota Izukawa MD.

Critical revision of manuscript for intellectual content by Hiroyuki Mushiake MD, PhD.

All members have been approved the final version to be published.

## Guarantor

Shota Izukawa MD and Hiroyuki Mushiake MD, PhD.

## Research registration number

Not applicable.

## Declaration of competing interest

We have nothing to declare in any categories.
